# COVID‐19 infection in patients with connective tissue disease: A multicity study in Hubei province, China

**DOI:** 10.1002/mco2.56

**Published:** 2021-02-04

**Authors:** Cong Ye, Jixin Zhong, Shaozhe Cai, Li Dong, Chuanjing Li, Xiaoqiang Hou, Xiaoqi Chen, Anbing Zhang, Wenli Chen, Dongchu He, Tao Zhou, Guilian Shang, Aichun Chu, Huiling Li, Qihuan Liu, Bin Wu, Xiangdong Yu, Tao Peng, Cheng Wen, Gang Hong Huang, Hao Huang, Qin Huang, Linchong Su, Wenping Chen, Huiqin Yang, Lingli Dong

**Affiliations:** ^1^ Department of Rheumatology and Immunology Tongji Hospital Tongji Medical College of Huazhong University of Science and Technology Wuhan Hubei China; ^2^ Jingzhou City Central Hospital of Hubei Province Jingzhou Hubei China; ^3^ Department of Rheumatology Xiaogan Hospital Affiliated to Wuhan University of Science and Technology Xiaogan Hubei China; ^4^ The First College of Clinical Medical Sciences China Three Gorges University Yichang Hubei China; ^5^ Department of Rheumatology Zhongnan Hospital of Wuhan University Wuhan Hubei China; ^6^ Department of Rheumatology and Immunology Xiangyang Central Hospital Xiangyang Hubei China; ^7^ Department of Rheumatology and Immunology Wuhan Central Hospital Wuhan Hubei China; ^8^ Department of Integrated Treatment General Hospital of Central Theater Command Wuhan Hubei China; ^9^ Department of Rheumatology and Immunology Wuhan Puren Hospital Affiliated to Wuhan University of Science and Technology Wuhan Hubei China; ^10^ Department of Rheumatology and Immunology Tianyou Hospital Affiliated to Wuhan University of Science and Technology Wuhan Hubei China; ^11^ Department of Prevention and Health Care Renmin Hospital of Wuhan University Wuhan Hubei China; ^12^ Department of Rheumatology and Immunology The Traditional Chinese Medical Hospital of Hubei Province Wuhan Hubei China; ^13^ Department of Rheumatology and Immunology Affliated Dongfeng Hospital Hubei University of Medicine Shiyan Hubei China; ^14^ Department of Rheumatology and Immunology The First People's Hospital of Jingzhou Jingzhou Hubei China; ^15^ Department of Rheumatology and Immunology Huangshi Central Hospital of Edong Healthcare Group Huangshi Hubei China; ^16^ Department of Rheumatology and Immunology Hankou Hospital of Wuhan Wuhan Hubei China; ^17^ Department of Endocrinology Xiaogan First People's Hospital Xiaogan Hubei China; ^18^ Department of Rheumatology and Immunology China Resources and Wisco General Hospital Wuhan Hubei China; ^19^ Department of Rheumatology and Immunology The First People's Hospital of Tianmen Tianmen Hubei China; ^20^ Department of Nephrology and Rheumatology Enshi Tujia and Miao Autonomous Prefecture Central Hospital Enshi Hubei China; ^21^ Department of Rheumatology and Immunology Minda Hospital of Hubei Minzu University Enshi Hubei China; ^22^ Department of Rheumatology and Immunology Huanggang Central HospitaI Huanggang Hubei China; ^23^ Department of Rheumatology and Immunology Wuhan No.1 Hospital Wuhan Hubei China

**Keywords:** connective tissue disease, COVID‐19, hydroxychloroquine, rheumatic disease, SARS‐CoV‐2

## Abstract

Novel Coronavirus disease 2019 (COVID‐19) has spread rapidly around the world. Individuals with immune dysregulation and/or on immunosuppressive therapy, such as rheumatic patients, are considered at greater risk for infections. However, the risks of patients with each subcategory of rheumatic diseases have not been reported. Here, we identified 100 rheumatic patients from 18,786 COVID‐19 patients hospitalized in 23 centers affiliated to Hubei COVID‐19 Rheumatology Alliance between January 1 and April 1, 2020. Demographic information, medical history, length of hospital stay, classification of disease severity, symptoms and signs, laboratory tests, disease outcome, computed tomography, and treatments information were collected. Compared to gout and ankylosing spondylitis (AS) patients, patients with connective tissue disease (CTD) tend to be more severe after COVID‐19 infection (*p *= 0.081). CTD patients also had lower lymphocyte counts, hemoglobin, and platelet counts (*p* values were 0.033, < 0.001, and 0.071, respectively). Hydroxychloroquine therapy and low‐ to medium‐dose glucocorticoids before COVID‐19 diagnosis reduced the progression of COVID‐19 to severe/critical conditions (*p *= 0.001 for hydroxychloroquine; *p *= 0.006 for glucocorticoids). Our data suggests that COVID‐19 in CTD patients may be more severe compared to patients with AS or gout.

## Graphical abstract

A total of 100 patients with rheumatic disease and COVID‐19 were collected from 23 centers in Hubei, China. COVID‐19 in patients with connective tissue disease was more severe compared to patients with ankylosing spondylitis or gout. Hydroxychloroquine therapy and low‐dose glucocorticoids before diagnosis of COVID‐19 reduced the progression of COVID‐19 to severe/critical conditions.

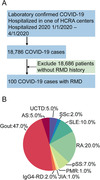

## 1 INTRODUCTION

The current pandemic of novel coronavirus disease 2019 (COVID‐19) is caused by severe acute respiratory syndrome coronavirus 2 (SARS‐CoV‐2) and has caused over 72 million laboratory confirmed cases and over 1.6 million deaths as of December, 2020, according to a report from Johns Hopkins University in the United States.^1^ Individuals with immune dysregulation and/or on immunosuppressive therapy, such as patients with rheumatic disease, are considered at greater risk for infections. Our recent studies confirmed that patients with rheumatic disease were at higher risks of COVID‐19 infection and higher risks of developing respiratory failure after COVID‐19 infection.^2^, ^3^ However, the risks of patients with each subcategory of rheumatic diseases have not been reported.

Most patients with rheumatic diseases, including systemic lupus erythematosus, Sjögren's syndrome, and rheumatoid arthritis, have immune dysregulation and require immunosuppressive medication and/or corticosteroids to prevent disease progression. It is well accepted that individuals on these therapies are susceptible to infections. Moreover, therapies of corticosteroids and immunosuppressive agents may cover the symptom of fever. Therefore, these patients may not present with fever after infections and rheumatic disease may share similar symptoms/signs with COVID‐19 (such as fever and low lymphocyte count), causing difficulties in COVID‐19 diagnosis in patients with rheumatic disease.

Interestingly, several antirheumatic drugs, including chloroquine, hydroxychloroquine (HCQ), and tocilizumab, are suggested to have antiviral effects against SARS‐CoV‐2,^4^, ^5^, ^6^, ^7^ although direct evidence is still lacking.^8^ It remains challenging to balance the needs of controlling rheumatic disease activity and risks of infections during the pandemic. In this study, we aim to analyze the clinical features of COVID‐19 in rheumatic patients and assess the effect of antirheumatic therapies on COVID‐19 severity. We identified 100 rheumatic patients from 18,786 laboratory confirmed COVID‐19 patients hospitalized in 23 centers affiliated to Hubei COVID‐19 Rheumatology Alliance between January 1 and April 1, 2020.

## 2 METHODS

### 2.1 Patient enrollment and study design

**FIGURE 1:**
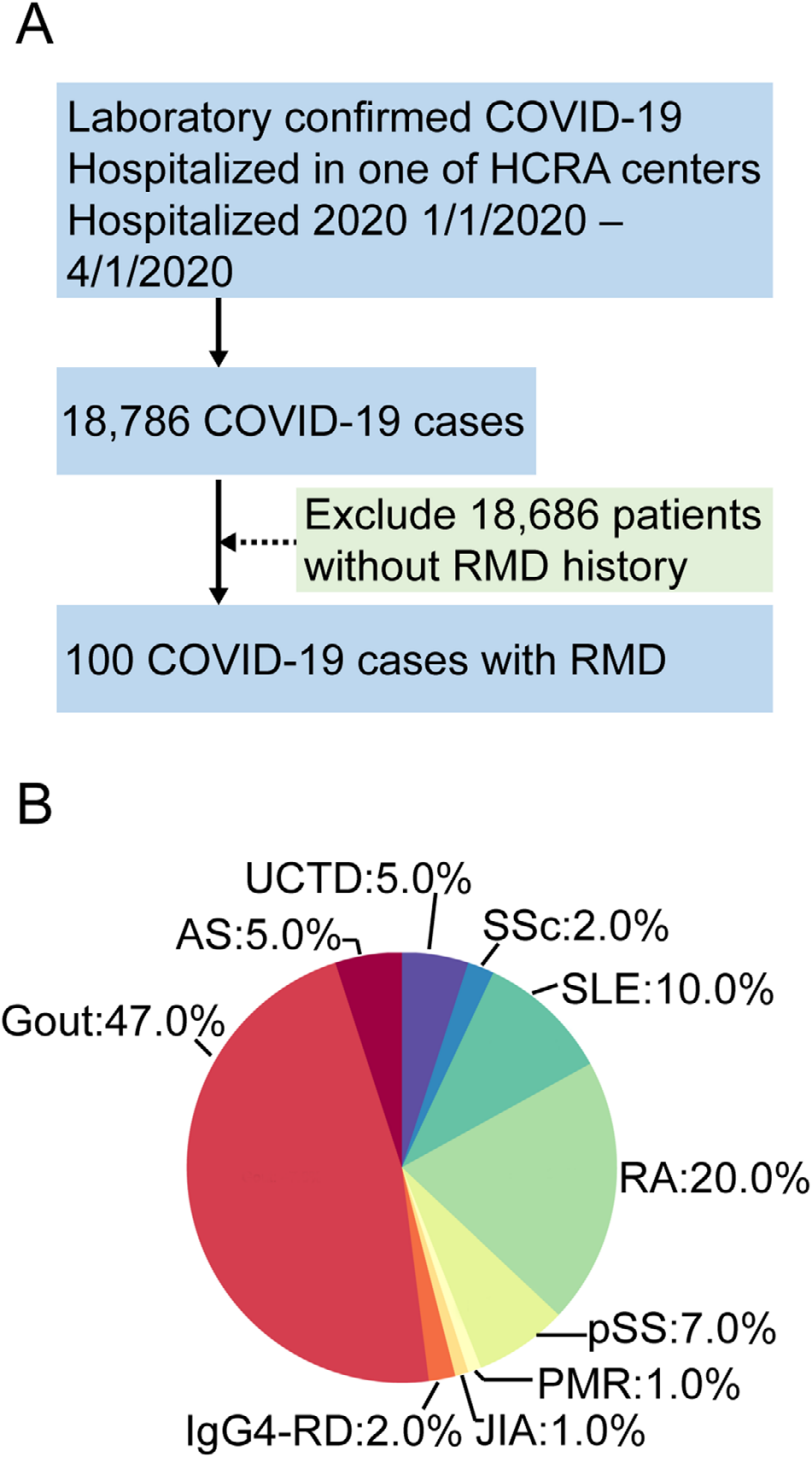
**Case screening**: A, Flow chart of cases screening. HCRA, Hubei COVID‐19 Rheumatology Alliance; RMD, rheumatic disease. B, Pie chart showing the disease composition of patients with COVID‐19 and rheumatic disease. AS, ankylosing spondylitis; PMR, polymyalgia rheumatica; pSS, primary Sjögren's syndrome; RA, rheumatoid arthritis; SLE, systemic lupus erythematosus; SSc, systemic sclerosis; IgG4‐RD, IgG4‐related disease; JIA, juvenile idiopathic arthritis; UCTD, undifferentiated connective tissue disease

In this multicenter retrospective study, a total of 100 COVID‐19 patients with rheumatic disease history were identified among 18,786 laboratory confirmed COVID‐19 cases, by searching the medical database in 23 tertiary hospitals affiliated to Hubei COVID‐19 Rheumatology Alliance (Figure 1A). All these patients were diagnosed with COVID‐19 according to China's Novel Coronavirus Pneumonia Diagnosis and Treatment Plan (7th Edition) and hospitalized between January 1 and April 1, 2020. The standards of clinical classifications, including mild, moderate, severe, and critically ill, were described earlier.^9^ This study was reviewed and approved by Tongji Hospital Medical Ethics Committee, Tongji Medical College of Huazhong University of Science and Technology (Approval # TJ‐IRB20200344). Written informed consent was waived because of the rapid spread of this emerging infection. This study was registered at chictr.org (registration # ChiCTR2000030795).

### 2.2 Data collection

Deidentified demographic data (gender and age), medical history, diagnosis, including disease severity, length of hospital stay, symptoms and signs, laboratory tests, disease outcome, computed tomography (CT), and treatments (including treatments for both rheumatic disease and COVID‐19), were obtained from electronic hospital information system. Two investigators (C.Y. and S.C.) independently reviewed and analyzed the data according to a standardized written protocol to ensure that data are accurate and consistent.

**Key Messages**

- Patients with connective tissue diseases tend to be more severe after COVID‐19 infection.
- Patients already on hydroxychloroquine and/or low‐ to medium‐dose glucocorticoid therapy may continue antirheumatic therapy during COVID‐19 pandemics.

### 2.3 Laboratory measurements of SARS‐CoV‐2

SARS‐CoV‐2 RNA in throat swab samples was determined by a real‐time reverse transcription polymerase chain reaction assay as described earlier.^3^ RNAs from the throat swab were extracted using a respiratory sample RNA isolation kit (Biogerm, Shanghai, China) in 2 h after sample collection. ORF1ab (forward primer sequence: CCCTGTGGGTTTTACACTTAA; reverse primer sequence: ACGATTGTGCATCAGCTGA) and nucleocapsid protein N (forward primer sequence: GGGGAACTTCTCCTGCTAGAAT, reverse primer sequence: CAGACATTTTGCTCTCAAGCTG) were amplified using a SARS‐Cov‐2 nucleic acid detection kit as instructed by manufacturer (Shanghai Bio‐germ Medical Technology Company).

### 2.4 Antibody detection

Circulating anti‐SARS‐CoV‐2 IgM/IgG antibodies in patients were detected by a chemiluminescence immunoassay kit designed for SARS‐CoV‐2 (Xiamen Innodx Biotech Co., Ltd), as instructed by the manufacturer.

### 2.5 Statistical analysis

Statistical analyses were performed by IBM SPSS® Software (version 25) or R software (version 3.6.2). Quantitative data were presented with median [IQR], categorical data were presented with number [percentage]. Mann–Whitney U test was used to compare the difference of quantitative data and ranked data, while χ^2^ test and Fisher's exact test were used to compare the proportions of categorical data. Pie plot was drawn using R. A *p* value less than 0.05 was considered as statistically significant.

## 3 RESULTS

### 3.1 Demography of rheumatic patients infected with COVID‐19

A total of 18,786 COVID‐19 cases were diagnosed through laboratory confirmation between January 1 and April 1, 2020 in 23 hospitals in Hubei, the COVID‐19 epicenter in China (Figure 1A). Among these COVID‐19 patients, there were 100 of them had rheumatic diseases, including 98 cases of COVID‐19 nucleic acid positive and 2 cases of nucleic acid negative but IgM/IgG double positive. According to the underlying immunopathology and treatment strategy, we divided these patients into three groups: connective tissue disease (CTD), ankylosing spondylitis (AS), and gout. There were 20 rheumatoid arthritis, 10 systemic lupus erythematosus, 7 Sjögren's syndrome, 5 undifferentiated CTD, 2 systemic sclerosis, 2 IgG4‐related disease, 1 juvenile idiopathic arthritis, and 1 polymyalgia rheumatic in CTD group (Figure 1B).

### 3.2 Clinical characteristics of COVID‐19 patients with medical history of AS, CTD, and gout

The number of females is about twice that of males in the CTD group, while the majority of the gout patients are males (Table 1). This is consistent with the gender characteristics of these two types of diseases. No significant difference in the age was noted among these three groups: the majority of the patients were middle‐aged and elderly, only nine of them under the age of 40 (9%). Among these 100 rheumatic patients, six died of COVID‐19 (6%): five cases of CTD (three cases of rheumatoid arthritis, one Sjögren's syndrome case, and one case of systemic lupus erythematosus) and one case of gout. The disease also seemed to be more severe in the CTD group: percentages of critically ill cases were 16.7%, 0%, and 2.1% for CTD, AS, and gout, respectively (*p *= 0.081; Table 2). This is probably because CTD patients have immune dysregulation and are usually on immunosuppressive mecication,^10^ and they should pay extra attention to personal protection during pandemic.

**TABLE 1:** Clinical characteristics of rheumatic patients infected with COVID‐19

	AS	CTD	Gout	
	*n* = 5	*n* = 48	*n* = 47	p*
Characteristics				
Age	49.0 [44.0, 55.0]	61.5 [47.7, 67.0]	58.0 [44.5, 67.0]	0.626
Sex (male/female)	2/3	15/33	43/4	<0.001
Duration of rheumatic disease (years)	8.5 [5.3, 11.2]	5.0 [3.0, 10.2]	3.0 [0.5, 3.2]	0.017
Complication				
HBP	1 (20.0)	7 (14.6)	15 (31.9)	0.079
CHD	0 (0)	2 (4.2)	11 (23.4)	0.015
DM	0 (0)	2 (4.2)	4 (8.5)	0.654
Intracranial hemorrhage	0 (0)	1 (2.1)	0 (0)	1.000
Cerebral infarction	0 (0)	3 (6.3)	1 (2.1)	0.625
Viral hepatitis	1 (20.0)	1 (2.1)	0 (0)	1.000
Bronchiectasia	0 (0)	2 (4.2)	0 (0)	0.495
COPD	0 (0)	3 (6.3)	1 (2.1)	0.625
ILD	1 (20.0)	4 (8.3)	4 (8.5)	1.000
Symptom				
Fever	4 (80.0)	40 (83.3)	35 (74.5)	0.419
Temperature (°C)	38.9 [38.6, 39.1]	38.4 [37.9, 39.0]	38.4 [38.0, 39.0]	0.97
Cough	2 (40.0)	31 (64.6)	35 (74.5)	0.410
Expectoration	0 (0)	21 (43.8)	11 (23.4)	0.060
Dyspnea	0 (0)	24 (50)	20 (42.6)	0.602
Fatigue	0 (0)	24 (50)	11 (23.4)	0.013
Diarrhea	0 (0)	9 (18.8)	15 (31.9)	0.215
Joint pain	0 (0)	12 (25.0)	2 (4.3)	0.010
Joint swelling	1 (20.0)	6 (12.5)	0 (0)	0.026
Back pain	1 (20.0)	0 (0)	0 (0)	1.000
Myalgia	0 (0)	1 (2.1)	0 (0)	1.000
Rash	0 (0)	2 (4.2)	0 (0)	0.495
Oral ulcer	0 (0)	1 (2.1)	0 (0)	1.000

Abbreviations: AS, ankylosing spondylitis; CHD, coronary heart disease; COPD, chronic obstructive pulmonary disease; CTD, connective tissue disease; DM, diabetes mellitus; HBP, hypertension; ILD, interstitial lung disease; WBC, white blood cell.

*p value was calculated by comparing values between CTD and gout.

**TABLE 2:** Clinical classification, treatment, and outcome of rheumatic patients infected with COVID‐19

	AS	CTD	Gout	
	*n* = 5	*n* = 48	*n* = 47	p*
Clinical classification of COVID‐19				0.081
Mild	3 (60.0)	15 (31.2)	18 (38.3)	
Moderate	2 (40.0)	12 (25.0)	17 (36.2)	
Severe	0 (0)	13 (27.1)	11 (23.4)	
Critically ill	0 (0)	8 (16.7)	1 (2.1)	
Ventilator	0 (0)	8 (16.7)	1 (2.1)	0.039
Flare of rheumatic diseases during hospitalization	1 (20.0)	7 (14.6)	14 (29.8)	0.124
Treatment for flare	0 (0)	7 (14.6)	0 (0)	
NSAIDs	1 (20.0)	4 (8.3)	12 (25.5)	
Glucocorticoid (dosage increased)	0 (0)	3 (6.3)	0 (0)	
Colchicine	0 (0)	0 (0)	2 (4.3)	
Glucocorticoids during hospitalization	0 (0)	30 (62.5)	14 (29.8)	
Death	0 (0)	5 (10.4)	1 (2.1)	0.215

Abbreviations: AS, ankylosing spondylitis; COVID‐19, coronavirus disease 2019; CTD, connective tissue disease; NSAIDs, nonsteroidal anti‐inflammatory drugs.

*p value was calculated by comparing values between CTD and gout.

Compared to gout and AS patients, CTD patients had lower lymphocyte counts, hemoglobin, and platelet counts (*p* values were 0.033, < 0.001, and 0.071, respectively; Table 3). The uric acid and creatinine levels were higher in the gout patients than those in the other two groups (*p *< 0.001 for both uric acid and creatinine; Table 3). Most patients with gout did not use uric acid‐lowering drugs before diagnosis, and nearly 30% of patients had acute attacks of gout during the hospital stay.

**TABLE 3:** Laboratory indices of rheumatic patients infected with COVID‐19

	AS	CTD	Gout	
	*n* = 5	*n* = 48	*n* = 47	p*
WBC (10^9^/L)	4.4 [4.0, 5.0]	5.5 [4.5, 7.4]	6.5 [4.7, 8.0]	0.128
Lym (10^9^/L)	1.2 [1.2, 1.4]	0.9 [0.6, 1.3]	1.2 [0.9, 1.6]	0.013
Neu (10^9^/L)	2.5 [2.5, 3.1]	3.9 [2.7, 5.2]	4.3 [2.8, 5.8]	0.363
Hb (g/L)	130.0 [119.0, 153.0]	114.5 [100.8, 132.3]	139.0 [123.0, 152.5]	<0.001
PLT (10^9^/L)	154.0 [120.0, 178.0]	181.0 [123.3, 237.8]	220.0 [168.5, 262.0]	0.037
ALT (U/L)	24.0 [24.0, 34.3]	25.0 [10.0, 54.0]	34.0 [20.0, 57.5]	0.085
AST (U/L)	26.0 [20.0, 33.0]	29.0 [20.0, 44.0]	30.4 [22.5, 43.0]	0.735
UA (μmol/L)	239.0 [180.0, 376.1]	254.0 [186.0, 297.6]	403.0 [290.0, 500.3]	<0.001
Cr (μmol/L)	67.0 [56.0, 70.2]	62.0 [52.5, 87.0]	88.0 [74.5, 107.4]	<0.001
PCT (ng/mL)	0.03 [0.02, 0.19]	0.10 [0.05, 0.28]	0.07 [0.05, 0.21]	0.67
CRP (mg/L)	5.0 [1.5, 20.0]	20.6 [5.2, 58.0]	21.7 [3.1, 56.5]	0.987
ESR (mm/h)	20.0 [15.0, 36.0]	39.0 [19.5, 53.5]	28.00 [10.0, 51.0]	0.303

Abbreviations: ALT, alanine aminotransferase; AS, ankylosing spondylitis; AST, aspartate aminotransferase; Cr, creatinine; CRP, C‐reactive protein; CTD, connective tissue disease; COVID‐19 , coronavirus disease 2019; ESR, erythrocyte sedimentation rate; Hb, hemoglobin; Lym, lymphocyte; Neu, neutrophil; PCT, procalcitonin; PLT, platelet; UA, uric acid; WBC, white blood cell.

*p value was calculated by comparing values between CTD and gout.

### 3.3 Patients with sustained use of HCQ in CTD patients before infection had less severe and critical cases of COVID‐19

**FIGURE 2:**
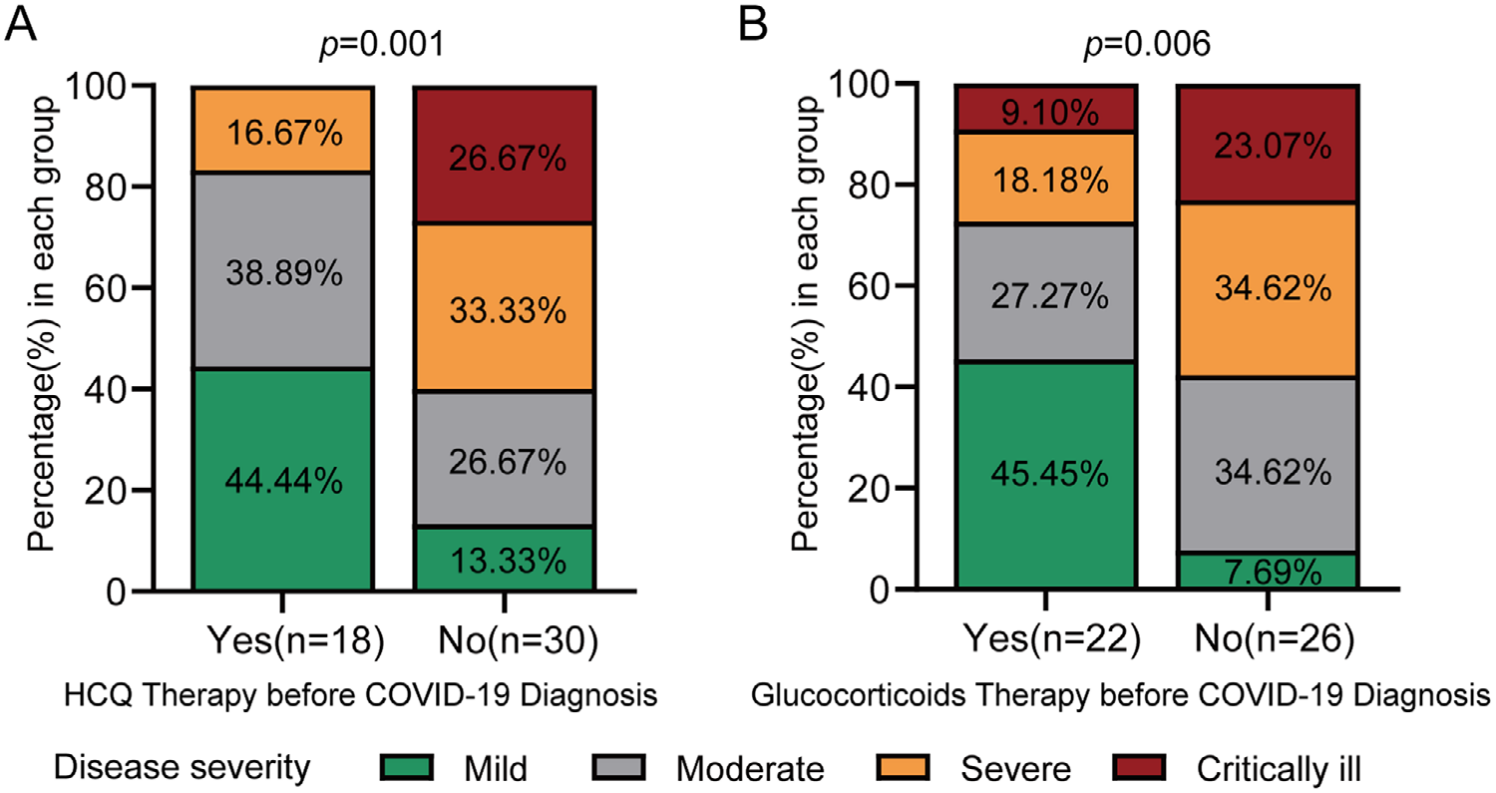
**COVID‐19 severity**: COVID‐19 severity in CTD patients with HCQ (A) or glucocorticoids (B) therapy before COVID‐19 diagnosis was shown

None of the patients in AS and gout group took chronic glucocorticoids or conventional antirheumatic drugs (Table 4). There were 37.5% CTD patients (18/48) using HCQ (0.2–0.4 g/day) before COVID‐19 diagnosis and three of them had severe or critical COVID‐19 conditions. In contrast, 18 out of the rest 30 patients without prior HCQ treatment had severe or critical conditions (*p *< 0.05). In addition, none of the five deceased patients were using HCQ before the diagnosis of COVID‐19. The potential therapeutic effects of HCQ on COVID‐19, especially in patients with disordered immunity, are still controversial.^5^, ^8^, ^11^ Our result indicates that sustained use of HCQ, in CTD patients at least, was associated with a lower proportion of severe/critical conditions (Figure 2A).

**TABLE 4:** Medication before the diagnosis of COVID‐19

Medication	AS (*n* = 5)	CTD (*n* = 48)	Gout (*n* = 47)
Glucocorticoids	0 (0)	22 (45.8)	0 (0)
Hydroxychloroquine	0 (0)	18 (37.5)	0 (0)
NSAIDs	2 (40.0)	7 (14.6)	0 (0)
Methotrexate	0 (0)	8 (16.7)	0 (0)
Leflunomide	0 (0)	7 (14.6)	0 (0)
Thalidomide	1 (20.0)	0 (0)	0 (0)
Tripterygium glycosides	0 (0)	3 (6.3)	0 (0)
Total glucosides of paeony	1 (20.0)	7 (14.6)	0 (0)
TNFi	2 (40.0)	0 (0)	0 (0)
Febuxostat	0 (0)	0 (0)	8 (17.0)
Allopurinol	0 (0)	0 (0)	3 (6.4)
Sodium bicarbonate	0 (0)	0 (0)	3 (6.4)

Abbreviations: AS, ankylosing spondylitis; CTD, connective tissue disease; NSAIDs, nonsteroidal anti‐inflammatory drugs.

### 3.4 Low‐ to medium‐doses of glucocorticoids in CTD patients before infection reduces the progression of COVID‐19 to severe and critical conditions

There were 45.8% CTD patients (22/48) who took low‐ to medium‐dose glucocorticoids (5–15 mg/day prednisone) before COVID‐19 diagnosis. Compared to those without glucocorticoids use before diagnosis, less patients on glucocorticoid therapy had severe/critical conditions (6/22 vs. 15/26, *p *< 0.05; Figure 2B). This result indicates that low‐ to medium‐dose glucocorticoids may reduce the progression of COVID‐19 from mild/moderate conditions to severe/critical conditions, and rheumatic patients already on low‐ to medium‐dose glucocorticoids are encouraged to continue glucocorticoid therapy during the pandemic. Two out of the five AS patients were on TNFα inhibitor treatment and both were diagnosed with mild illness.

Our data provides important information for the guidance of antirheumatic medication during pandemics. Rheumatic patients already on HCQ and/or low‐ to medium‐dose glucocorticoid therapy may continue their antirheumatic therapy during COVID‐19 pandemics, although this is a multicenter retrospective study and further investigation may be required to warrant these conclusions.

## 4 DISCUSSION

Angiotensin‐converting enzyme 2 (ACE2) mediates the entry of SARS‐CoV‐2, the recently identified human coronavirus,^12^ into host cells. The spike protein on SARS‐CoV‐2 has a high binding affinity to human ACE2, suggesting a high level of infectivity.^13^, ^14^

In this case series, we found that CTD patients tend to be more severe when infected with COVID‐19 as demonstrated by the finding that more CTD patients had critical condition and more CTD patients died of COVID‐19 compared to AS and gout patients. This is probably because CTD patients have immune dysregulation and are usually on immunosuppressive mecication,^10^ considering the importance of antiviral immunity in SARS‐CoV‐2 clearance. Antiviral immune response plays an important role in the fighting against COVID‐19. Most, reported to be 80.9% in China,^15^ COVID‐19 patients presented with mild symptoms and eventually recovered completely due to the effective antiviral immune response. Recovered COVID‐19 patients showed an elevation of antibody‐secreting cells and activated follicular helper T cells a few days before the symptomatic recovery and the clearance of SARS‐CoV‐2.^16^ These two subsets of immune cells are critical effector cells in antiviral immunity, whose increase indicates that antiviral immunity is important for the clearance of SARS‐CoV‐2. Thus, patients with rheumatic disease should pay extra attention to personal protection during pandemic, as they might have an elevated risk of severe COVID‐19.

Hyperactivation of immune responses, on the other hand, may cause serious damage to host tissues in COVID‐19 infection. SARS‐CoV‐2 has been shown to impair the functionality of immune cells in critically ill patients. These patients showed reduced lymphocyte counts, especially CD4^+^ helper T cells and CD8^+^ cytotoxic T lymphocytes. IFNγ‐expressing helper T cells also reduced in severe cases of COVID‐19.^17^ Studies indicate that severe cases of COVID‐19 displayed hyperinflammatory status, accompanied by an increase of multiple tissue damage markers and multiorgan failure.^18^, ^19^ Thus, many immunosuppressive agents, such as interleukin‐6 blocker tocilizumab, glucocorticoids, and antimalaria agent HCQ, have been tested for their therapeutic potential in COVID‐19. HCQ is a widely used immunosuppressant in rheumatic disorders.^20^, ^21^ It is a derivate of chloroquine and exerts immunomodulatory effects via interfering Toll‐like receptor signaling and activity of cyclic GMP‐AMP synthase, resulting in the suppression of immune cell activation and release of inflammatory cytokines.^22^ In addition, both chloroquine and its derivative HCQ have shown antiviral potentials. HCQ can inhibit NS2B‐NS3 protease of Zika Virus and significantly decrease Zika virus infection in placental cells.^23^ Chloroquine is reported to affect terminal glycosylation of SARS‐CoV‐2 entry receptor ACE2.^14^, ^24^ Furthermore, chloroquine was also able to elevate endosomal pH and thus reduce virus/cell fusion. It has been reported that both chloroquine and HCQ were able to suppress SARS‐CoV‐2 infection in an *in vitro* experiment.^25^, ^26^ Gautret and coauthors have also recently shown that HCQ was able to accelerate SARS‐CoV‐2 clearance in a randomized trial with 22 COVID‐19 patients.^5^ In the current report, we found that the use of HCQ in rheumatic patients was associated with a lower proportion of severe/critical conditions. However, it should be noted that this conclusion is based on the comparison with rheumatic patients using other immunosuppressive agents. It remains unclear whether chloroquine and HCQ may protect individuals who do not need to use immunosuppressant from COVID‐19.

In our study, we also found an association between the use of low‐ to medium‐dose glucocorticoid and lower proportion of severe/critical conditions of COVID‐19 in patients with rheumatic diseases. Glucocorticoids are steroid hormones produced in adrenal cortex. They are commonly used in patients with rheumatic disease due to the rapid immunosuppressive actions.^27^ Glucocorticoids have been used to treat severe case of coronavirus infection, including COVID‐19, to suppress the hyperinflammatory response.^28^, ^29^ However, their use in such diseases has been controversial.^30^ Unexpectedly, we found that less patients on low‐ to medium‐dose glucocorticoid therapy had severe/critical COVID‐19 conditions, compared to those without glucocorticoids use before COVID‐19 diagnosis. This is probably because the dose of glucocorticoids in these patients was relatively low (equals to 5–15 mg/day prednisone) and there has been research reporting that low‐ to medium‐dose glucocorticoids does not increase the risk of infection.^31^, ^32^ This result indicates that rheumatic patients already on low‐ to medium‐dose glucocorticoids may continue glucocorticoid therapy during the pandemic. In summary, our data provide important information for the guidance of antirheumatic medication during pandemics. Patients with CTD might have a greater risk of severe COVID‐19 when infected. Although patients already on HCQ and/or low‐ to medium‐dose glucocorticoid therapy seem to have a lower incidence of severe/critical COVID‐19 conditions, extra caution should be taken as their dysregulated immune responsiveness and use of immunosuppressive agents. There were several limitations within this investigation. First, this study retrospectively analyzes clinical data of COVID‐19 patients with a medical history of rheumatic diseases and there might be biases that are commonly seen in retrospective investigations. Second, the sample size is not large enough although we included 23 medical centers in Hubei province. Further investigation with a larger sample size may be required to warrant these conclusions. Third, we did not evaluate the potential effects of biological agents, such as TNFα inhibitors in COVID‐19, due to the limited number of cases with biological agent usage.

## CONFLICTS OF INTEREST

We declare no competing interests.

## ETHICS APPROVAL

This study was reviewed and approved by Tongji Hospital Medical Ethics Committee, Tongji Medical College of Huazhong University of Science and Technology (Approval # TJ‐IRB20200344). This study was registered at chictr.org (registration # ChiCTR2000030795).

## AUTHOR CONTRIBUTIONS

All authors contributed substantially to this work. L.D. (Lingli Dong), H.Y., W.C. (Wenping Chen), and J.Z. designed the study, performed data analysis, and wrote the manuscript. C.Y., J.Z., and S.C. analyzed the data and drafted the manuscript. C.Y., L.D. (Li Dong), C.L., X.H., X.C., A.Z., W.C. (Wenli Chen), D.H., T.Z., G.S., A.C., H.L., Q.L., B.W., X.Y., T.P., C.W., G.H.H., H.H., Q.H., and L.S. were responsible for the collection and interpretation of data. All the authors participated in this study and approved the final version of the article.

## Data Availability

All relevant data from this study are included in the article.
